# Safety and efficacy of fecal microbiota transplantation in the treatment of Parkinson’s disease: a systematic review of clinical trials

**DOI:** 10.3389/fnins.2025.1639911

**Published:** 2025-08-20

**Authors:** Kewei Chen, Lirong Sun, Yilan Liu, Ran Chen

**Affiliations:** ^1^Department of Neurology Shaoxing Hospital of Traditional Chinese Medicine, Shaoxing TCM Hospital Affiliated to Zhejiang Chinese Medical University, Shaoxing, China; ^2^Shaoxing City Keqiao District Hospital of Traditional Chinese Medicine, Shaoxing, China

**Keywords:** Parkinson’s disease, fecal microbiota transplantation, microbiota, review–systematic, efficacy and safety

## Abstract

**Introduction:**

Parkinson’s disease (PD) is the second most common neurodegenerative disease with limited treatment options and increasing incidence. The Microbiota–Gut–Brain Axis (MGBA) offers new insights for PD treatment, as gut microbiota imbalances are linked to PD. Fecal microbiota transplantation (FMT) shows potential to improve gut dysbiosis and has gained attention for PD treatment.

**Methods:**

We conducted a review following PRISMA 2009 guidelines, searching PubMed, EMBASE, Web of Science, and Scopus up to December 1, 2024. We included clinical trials of FMT for PD patients, regardless of stage or type, with outcomes related to efficacy or safety. Non-clinical trials were excluded. Two investigators independently assessed studies, extracted data, and evaluated risk of bias and quality.

**Results:**

A total of 1,147 articles were retrieved, and six studies involving 104 patients were included. Four were randomized controlled trials, one was a cohort study, and one was a case series. Patients had a mean age of 63.2 years and disease duration of 5.6 years. After FMT, some patients showed improvements in UPDRS scores, H-Y grades, NMSS scores, and constipation symptoms, but results varied across studies. No serious FMT-related adverse events occurred. Most were mild gastrointestinal issues. Gut microbiota diversity and beneficial bacterial abundance changed after FMT, correlating with clinical outcomes. FMT materials were mostly from unrelated donors with diverse preparation and delivery methods.

**Discussion:**

FMT shows efficacy and safety in PD treatment but is insufficient as a standard due to study heterogeneity and small sample sizes. Future research needs larger samples, unified tools, and standardized FMT procedures. Combining FMT with other therapies may improve efficacy.

## 1 Introduction

Parkinson’s disease (PD) is a common clinical disorder characterized by loss of nigrostriatal dopaminergic neurons and abnormal folding and aggregation of intracellular *α*-synuclein in neurons. The incidence of PD is the second highest among the degenerative diseases of the central nervous system (CNS) and is on the rise (Ben-Shlomo et al., 2024). It is predicted that the number of PD patients worldwide will double to more than 10 million in 20 years (Dorsey and Bloem, 2018). The clinical symptoms of Parkinson’s disease mainly include resting tremor, muscle rigidity, bradykinesia, and postural gait disturbances. In addition to the typical motor symptoms, patients with PD clinically exhibit gastrointestinal symptoms—especially constipation—which may even precede the onset of motor symptoms. Currently, dopamine modulators are the first-line therapeutic agents for PD. However, the therapeutic efficacy is limited and may cause serious side effects (Armstrong and Okun, 2020). Therefore, there is a need to discover safe and effective treatments to address the increasing burden of PD in an aging population.

Recent studies have demonstrated the existence of a bidirectional communication system between the brain and gut, the MGBA, providing insights into the etiology and physiology of neurological disorders (Claudino Dos Santos et al., 2023). In recent years, an increasing number of reports have demonstrated that the composition of gut flora in PD patients differs from that of healthy individuals and that there is a state of flora imbalance. The presence of *α*-synuclein (αSyn) structures in the colon of individuals with early PD has been found, suggesting that the disease may first originate in the gut (Fricova et al., 2020). Microbiota transplantation from PD patients exacerbates neurological damage in a mouse model of αSyn overexpression (Sampson et al., 2016). The gut microbiota of PD patients exhibits marked dysbiosis compared to healthy controls, characterized by a depletion of anti-inflammatory properties (e.g., Blautia, Coprococcus, and Roseburia) and a concurrent expansion of pathobionts (Salim et al., 2023). For example, butyric acid-producing bacteria associated with anti-inflammatory pathways, such as Blautia, Coprococcus and Roseburia, are relatively less abundant in the faeces of PD patients. In particular, numerous studies have shown that disease severity in PD patients is associated with altered abundance of specific flora (Chen et al., 2022; Bedarf et al., 2017; Scheperjans et al., 2015).

In recent years, the treatment of patients with neurodegenerative diseases with fecal bacterial transplantation has received increasing attention. Positive results have been found for fecal microbiota transplantation (FMT) for Alzheimer’s Disease (Li et al., 2023), as well as promising results in the improvement of Alzheimer’s Disease symptoms with antibiotics (Bello-Medina et al., 2022). Early animal models reported positive results with FMT for PD (Zhao et al., 2021; Sun et al., 2018). The use of probiotics can alleviate the symptoms of constipation in PD (Scheperjans et al., 2015). These studies have generated interest in the use of FMT to reverse gut ecological dysregulation in PD patients to potentially alleviate symptoms. The aim of this systematic review is to review the current literature on FMT for the treatment of PD, focusing on the clinical efficacy, safety and different therapeutic approaches to FMT treatment.

## 2 Materials and methods

We conducted this systematic review following the principles outlined in the PRISMA 2009 guidelines (Moher et al., 2009).

### 2.1 Search strategy and study selection

We searched for clinical trial studies on FMT interventions tailored to treat symptoms in patients with Parkinson’s disease. The search was conducted up to 1st December 2024. Four electronic databases were searched: PubMed, EMBASE, Web of Science, and Scopus. Keywords used were ‘fecal’, ‘microbiota’, ‘transplantation’ and ‘Parkinson’s disease’, a total of 608 articles were retrieved. The inclusion criteria were as follows: (1) clinical trials at any stage (with or without a control group); (2) patients with any type of Parkinson’s disease; (3) the intervention had to be a microbiota transplantation; (4) Use placebo, standard treatment, or alternatives in control groups; (5) At least an outcome related to efficacy or safety should be reported. We excluded trials that did not utilize FMT and omitted non-clinical research (e.g., animal studies, *in vitro* studies, reviews, correspondence, editorials, news articles, and books).

The shortlisted studies and articles were then thoroughly assessed by reading the full text. Two investigators (Chen K and Sun L) independently evaluated the title and abstract of each study. If any disagreement arose, it was submitted for consideration through discussion or consultation with the Senior Investigator (Chen R). The review was not registered.

### 2.2 Data extraction and quality assessment

Two researchers (Chen K and Sun L) independently conducted the study selection, data collection, and quality evaluation using predefined templates. Relevant clinical details were extracted from each eligible study, where possible, including details of the principal investigator, the year of publication, the geographical location, the research methodology, the demographics and characteristics of the participants, the criteria for the condition being studied (with a focus on its severity), the specifics of the intervention and its implementation (such as the dosage, frequency, administration route, treatment duration, and preparation of the FMT material), the primary and secondary outcomes, the follow-up period, the changes in the microbiome after FMT, the recorded adverse events (including deaths, hospitalizations, and other adverse incidents as defined by each study), and information about the donors.

### 2.3 Risk of bias and quality assessment

Bias was assessed in randomized controlled trials (RCTs) using the Cochrane Risk of Bias Tool (Higgins et al., 2011). Risk of bias in cohorts and case studies was assessed using the National Heart, Lung and Blood Institute Quality Assessment Tool (NHLBI Study Quality Assessment Tools, 2020). Further description of the risk of bias and quality assessment is provided in Supplementary Tables 1–3.

## 3 Results

**Figure 1:**
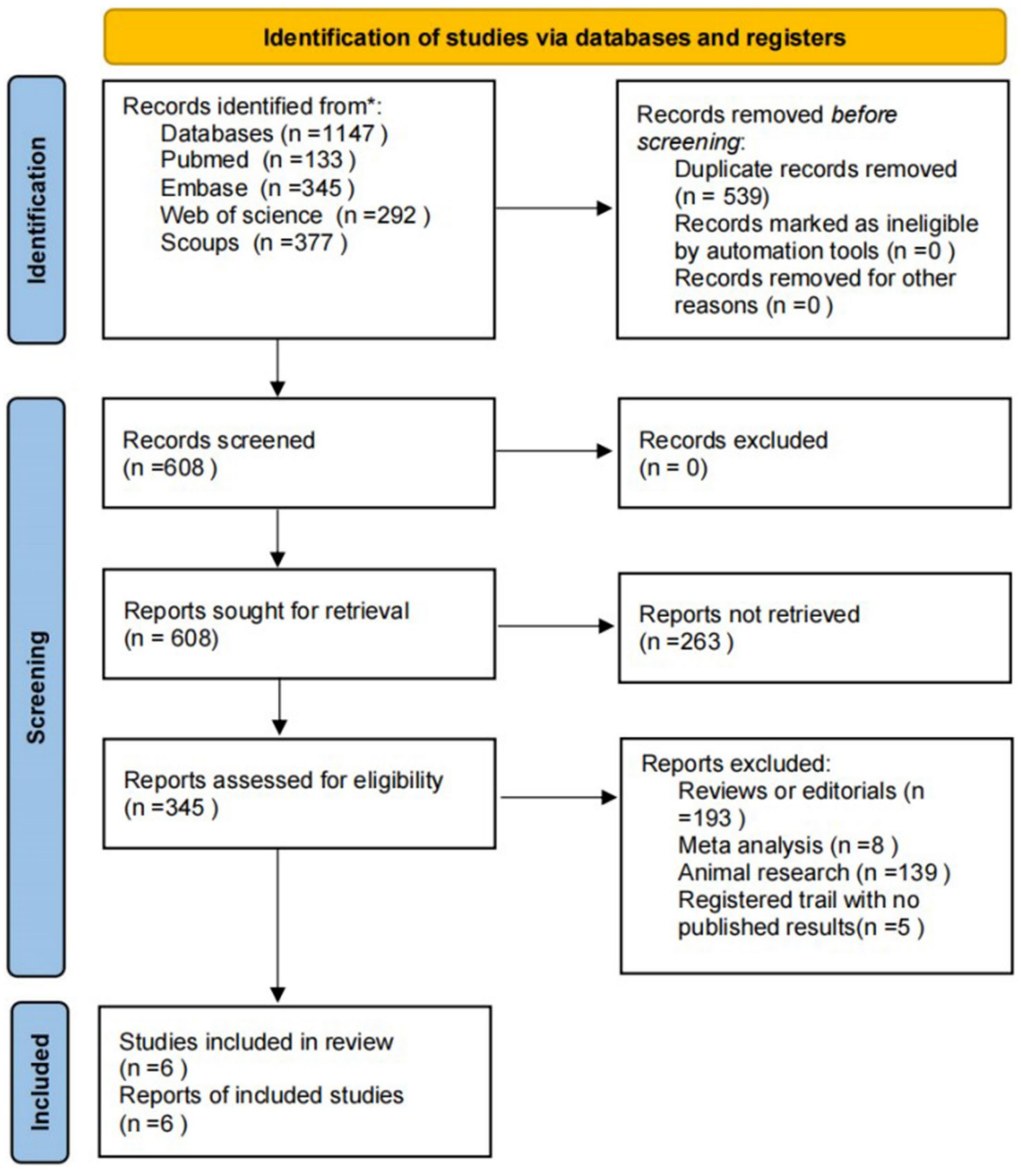
Literature retrieval and selection. Flowchart depicting the identification of studies via databases and registers. It starts with 1,147 records identified from databases and removes 539 duplicates. After screening 608 records, none are excluded. From 345 reports assessed, 263 are not retrieved, and 345 assessed for eligibility. After exclusions, six studies are included in the review.

The initial search yielded 1,147 publications. Following the removal of duplicates, 608 articles were screened for title and abstract. Subsequently, 345 full-text articles were examined for eligibility. Excluded were 193 reviews, 8 meta-analyses, 139 animal studies, and 5 ongoing clinical trials, resulting in six studies being included in the analysis (Segal et al., 2021; Kuai et al., 2021; Cheng et al., 2023; DuPont et al., 2023; Bruggeman et al., 2024; Scheperjans et al., 2024) (Figure 1). Due to significant heterogeneity among the included studies in interventions, participants, and outcome measures, we did not perform a meta-analysis. Differences in FMT administration routes, dosages, frequencies, donor screening criteria, and preparation methods, along with variations in patient populations and assessment tools (e.g., UPDRS, H-Y grade, NMSS) at inconsistent time points, made direct comparison and pooling of results challenging. These differences could obscure true study differences and lead to misleading conclusions, so a qualitative analysis was deemed more appropriate.

### 3.1 Patient characteristics and study type

A total of 104 patients received FMT treatment in the six included studies (Table 1). Four were randomized controlled studies, one cohort study and one case series study. The four randomized controlled studies followed the principle of random allocation, with placebo in the control group and FMT in the experimental group. Segal et al. conducted a case report of only six patients with PD who underwent FMT (Segal et al., 2021). Kuai et al., (2021) conducted a clinical trial comparing the symptoms of 11 patients before and after treatment with FMT. Chen et al. was a randomized, placebo-controlled trial but the follow up period was only 12 weeks (Cheng et al., 2023). DuPont et al. was a randomized, double-blind, placebo-controlled clinical trial, but only 12 individuals were included in the trial sample (DuPont et al., 2023). Bruggeman et al. (2024) and Scheperjans et al. (2024) conducted a randomized, double-blind, placebo-controlled clinical trial. Bruggeman et al. (2024) study was a phase II trial with a 12-month follow-up. There is no consistency in the definition and severity of PDD. Some studies focused on comparing FMT before and after treatment (Segal et al., 2021; Kuai et al., 2021; DuPont et al., 2023), while others focused on differences between the FMT and placebo groups (Table 2) (Cheng et al., 2023; Bruggeman et al., 2024; Scheperjans et al., 2024). In addition, the comparisons differed in terms of the metrics used, with most studies using PD-related assessment scales for disease symptoms, whereas DuPont et al. (2023) used only visual analog scales. As the six studies included did not assess the clinical outcome of FMT at the same time point and did not use the same methodology to assess disease activity, a proper meta-analysis could not be performed.

**Table 1:** Characteristics of PD patients treated with FMT.

Total number of studies, *n*	6
Total patient population, *n*	104
Study type. RCT/non-RCT, *n* (*n* = 104)	87/104
Male/Female patients, *n* (*n* = 104)	61/43
Mean age of patients, years (range) (*n* = 104)	63.2 (40–83)
Duration Parkinson’s, years (range) (*n* = 104)	5.6 (1–12)

**Table 2:** Clinical effects of FMT treatment to PD patients.

Author and year	Study type	Patient population	Sample size	The FMT preparation	Route	Outcomes	Follow-up
Segal et al. (2021)	Case series	3 male vs. 3 femal	6 FMT	Fresh stool (65 g) from each donor was immediately mixed in a blender with 300 mL of 0.9% sterile saline for several seconds until it developed a smooth consistency. The stool suspension was then filtered through a gauze pad to remove large particles. The stool suspension was mixed with glycerol (final concentration of 10%). 50 mL aliquots of the mixed suspension were prepared and frozen in − 80 ◦C to create a stool bank.	Colonoscopy	UPDRS-III, NMSS, Wexner constipation and BSS scores	2, 4, 8, 12, 16, 20, and 24 weeks
Kuai et al. (2021)	Cohort (open-label pilot study)	7 male vs. 4 femal	11 FMT	Frozen fecal microbiota was obtained from the China fmtBank (Nanjing, China)	Nasojejunal	the Hoehn-Yahr (H-Y) grade, UPDRS score, NMSS, PAC-QOL score and Wexner constipation score	12 weeks
Cheng et al. (2023)	RCT with open-label follow-up	32 male vs. 22 femal	27 FMT vs. 27 placebo	For stool donation for this trial, each donor provided stools for making FMT capsules for about 7 patients, and each patient in the FMT group was given 16 FMT capsules at each time, which are made from approximately 50 g of donated stool	Oral	the MDS-UPDRS score, safety (adverse effects), and evaluation for gastrointestinal disorders (including the IBS-SSS, GSRS, Bristol stool form scale, and IBS QOL scale scores) and evaluation for mental health (including the PHQ-9 scale, GDS-15 scale, GAD-7 scale, Montreal Cognitive Assessment, Mini-mental State Examination scores), and alterations in gut microbiota	4, 8 and 12 weeks
DuPont et al. (2023)	RCT with open-label follow-up	9 male vs. 3 femal	8 FMT vs. 4 placebo	We administer a dose of 100 g of donor feces,lyophilized to 1.5 g of powder, contained in 10 capsules	Oral	safety(adverse effects), Microbiome changes, Self-reported clinical global improvement using a 100 point visual analog scale	4, 8 and 12 weeks
Bruggeman et al. (2024)	RCT with open-label follow-up	29 male vs. 17 femal	22 FMT vs. 24 placebo	The faecal product was diluted with sterile saline and subsequently homoge nized anaerobically and filtered using a stomacher. Glycerol (10%) was added as a cryoprotectant to the filtered product resulting in a total volume of 200 mL. The faecal suspension was stored at −80 ◦C	Nasojejunal	the MDS-UPDRS score, Radiopaque pellets test, Levodopa-equivalent daily dose(LEDD), NMSS score, Parkinson’s Disease Quality of Life Questionnaire (PDQ-39), Wexner Constipation Scale, Geriatric Depression Scale(GDS), Parkinson Anxiety Scale (PAS), Lille Apathy Rating Scale(LARS), Parkinson’s Disease Sleep Scale(PDSS), Parkinson’s Fatigue Scale(PFS), Montreal Cognitive Assessment(MoCA)	3,6, 12 months
Scheperjans et al. (2024)	RCT with open-label follow-up	25 male vs. 20 femal	30 FMT vs. 15 placebo	Active treatment was a freeze-stored prepara tion of 30 g of feces from 1 of 2 donors, mixed with 150 mL of sterile physiological saline and 20 mL of 85% glycerol for cryoprotection to improve viability of microbes	Colonoscopy	the MDS-UPDRS score, the Hoehn-Yahr (H-Y) grade, Levodopa-equivalent daily dose(LEDD), TUG test (off medication), MoCA score, NMSS score, PDQ-39 SI score, IBS-SSS score, BDI-II score, BAI score, Wexner constipation score, Bowel movements, Intestinal volumes, Retained ROM markers	6, 12 months

### 3.2 The efficacy of FMT in the treatment of PD

**Figure 2:**
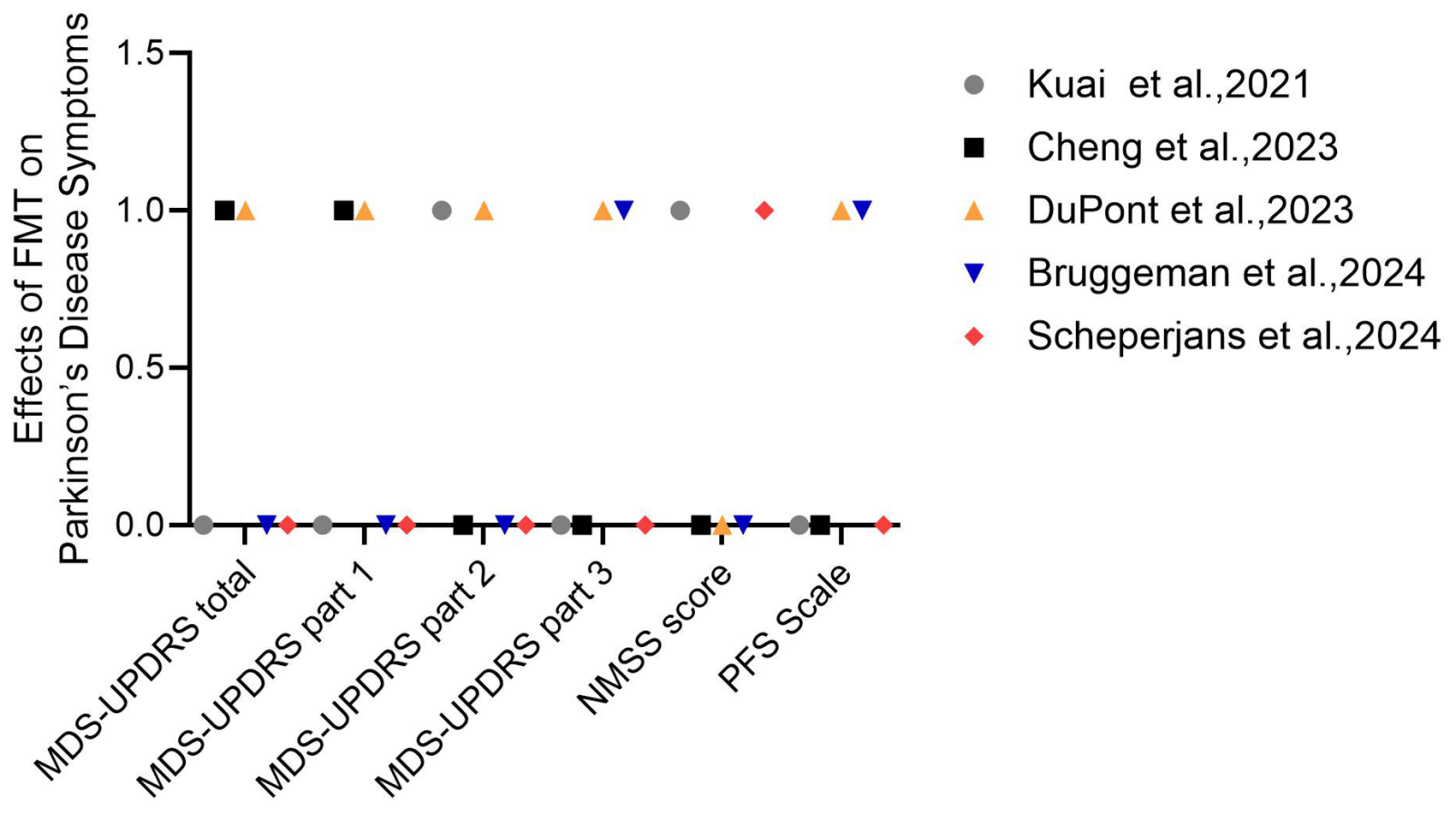
Effects of FMT on Parkinson’s Disease Symptoms. Changes in symptom scores in Parkinson’s disease patients after FMT. 1 = significant improvement, 0 = no statistically significant difference.

The Parkinson’s Disease Rating Scale (UPDRS) score and the Hoehn-Yahr (H-Y) Grade are overall indicators for assessing the severity of Parkinson’s disease. Kuai et al. (2021) reported a significant reduction in UPDRS score and H-Y score in PD patients after FMT treatment. However, the H-Y scores of PD patients did not change during the course of treatment in a case report by Segal et al. (2021). In a randomized controlled study by Cheng et al., UPDRS scores were essentially the same in both groups during the initiation phase, and after 3 months of treatment, total MDS-UPDRS total scores in the FMT group showed a greater decrease than with conventional PD treatment, suggesting that the clinical symptomatic improvement was more pronounced with FMT treatment (Cheng et al., 2023). However, in two other RCT studies (Scheperjans et al. and Bruggeman et al.), the improvement in MDS-UPDRS total scores were not significantly different between the two groups (Bruggeman et al., 2024; Scheperjans et al., 2024). For motor symptoms, this consisted mainly of MDS-UPDRS part 2 and part 3 scores (UPDRS-II and UPDRS-III scores). Kuai et al. (2021) demonstrated a significant improvement in UPDRS-II scores after 12 weeks of FMT treatment. Segal et al. (2021) reported a slight improvement in UPDRS-III scores in patients at 24 weeks. DuPont et al. (2023) found that after 4, 8 and 12 weeks of FMT treatment, motor deficits were significantly improved in PD patients compared to the placebo group. Of the randomized controlled studies, only the study by Bruggeman et al. (2024) found significant improvement in MDS-UPDRS part 3 (off-medication) scores compared to controls, and the scores changed most between 6 and 12 months (Figure 2).

For non-motor symptoms, the main assessment methods include the UPDRS-I score, the Non-Motor Symptoms Questionnaire (NMSS), the Geriatric Depression Scale (GDS-15), Parkinson’s Anxiety Scale (PAS), Riehl’s Apathy Rating Scale, Parkinson’s Disease Sleepiness Scale (PDSS), Parkinson’s Fatigue Scale (PFS), and Montreal Cognitive Rating Scale (MoCA), etc. The study by Segal et al. (2021) showed improvement in NMSS scores in 5 out of 6 patients 4 weeks after FMT. Kuai et al. (2021) showed significant reduction in NMSS after 6 and 12 weeks of FMT. For comparison between the two groups, Cheng et al. demonstrated a significant reduction in MDS-UPDRS-I in the FMT group compared to the control group at week 12 (Cheng et al., 2023). Scheperjans et al. (2024) demonstrated that the FMT group also had a significant improvement in non-motor symptom scores (NMSS scores); Bruggeman et al. (2024) found that the FMT group’s Parkinson’s Fatigue Scale (PFS) were significantly improved in the FMT group compared to both control groups.

Constipation symptoms were assessed using the Patient with Constipation Quality of Life Scale (PAC-QOL) and the Wexner constipation score. Segal et al. (2021) demonstrated that Wexner scores improved in 5/6 patients at 24 weeks after FMT. Kuai et al. (2021) demonstrated that PAC-QOL and Wexner scores improved after 12 weeks of FMT treatment. In addition, DuPont et al. (2023) found that subjective constipation scores improved in PD patients at 4, 8 and 12 weeks of FMT treatment. Multiple studies have demonstrated increased stool frequency and improved Bristol (BSS) scores following FMT treatment (Segal et al., 2021; Cheng et al., 2023; Scheperjans et al., 2024). Cheng et al. (2023) showed a significant increase in stool frequency (number of times per week) in the FMT group compared to the control group. Improvement in the number of complete bowel movements and spontaneous complete bowel movements after treatment was also significantly better in the FMT group than in the placebo group in the study by Scheperjans et al. (2024). However, there was no statistically significant difference in Bristol (BSS) scores between the two groups in these studies. In addition, Cheng et al. (2023) reported a significant improvement in the Irritable Bowel Syndrome-Severity Scale (IBS symptom severity scale, IBS-SSS) and Irritable Bowel Syndrome-Quality of Life (IBS-QOL) in the FMT group compared to the control group. Two studies evaluated the patients’ equivalent daily dose of levodopa (Levodopa-equivalent daily dose, LEDD) in clinical trials in which fecal transplants were performed. It was found that the phase LEDD increased over the course of the experiment in both the FMT and placebo groups (Bruggeman et al., 2024; Scheperjans et al., 2024). Scheperjans et al. (2024) demonstrated a significant increase of LEDD in the placebo group compared to the FMT group at 6 months.

### 3.3 The safety of FMT treatment

FMT-related fatalities and side effects leading to discontinuation of treatment have not been reported. Several studies reported minor self-limiting adverse events (Kuai et al., 2021; Cheng et al., 2023; DuPont et al., 2023; Bruggeman et al., 2024; Scheperjans et al., 2024). Most of these were gastrointestinal adverse events, including bloating, abdominal pain, nausea, diarrhea or constipation (Table 2). Gastrointestinal discomfort mostly occurs in the first week (Bruggeman et al., 2024). One RCT study reported significantly more side effects in the FMT group than in the control group (Scheperjans et al., 2024). Only one patient was reported by Segal et al. to have a serious adverse event requiring admission for observation-vasovagal syncopal episode 24 h after FMT (Segal et al., 2021).

### 3.4 The changes in the microbiome after FMT

Three studies assessed bacterial alpha and Beta diversity in faeces after FMT treatment. Two studies used 16S rDNA sequencing to analyze the microbiota (Kuai et al., 2021; Cheng et al., 2023), while another performed Whole Metagenome Shotgun sequencing on fecal samples (DuPont et al., 2023). All these studies employed analyses at the phylum, family and genus levels to assess the composition of fecal bacteria (Table 3). Kuai et al. (2021) found that bacterial alpha diversity was significantly increased in patients who experienced better clinical results after FMT treatment. Among them, Collinsella, Eubacterium_hallii, Ruminococcus_1, Dorea, and Blautia became the dominant genera during FMT treatment. DuPont et al. found that the genera that became dominant during FMT treatment were Roseburia and Colinsella (Table 3) (DuPont et al., 2023). Notably, a study by Kuai et al. (2021) found that the abundance and diversity indices at 12 weeks after FMT were not significantly different from normal. Two studies observed significant changes in Beta diversity after FMT treatment, with changes in the abundance of some specific bacteria (Kuai et al., 2021; DuPont et al., 2023). Kuai et al. (2021) found that Faecalibacterium, Blautia, Bacteroides, and Escherichia-Shigella increased significantly after FMT treatment, and changes in these flora correlated with clinical outcomes. DuPont et al. (2023) found Beta diversity in the significant difference between FMT-treated and placebo-treated groups. After 13 weeks of treatment, Lactobacillaceae, Limnochordaceae, and Peptostreptococcaceae were significantly more abundant in FMT group. In addition, Cheng et al. (2023) found that the genera of bacteria in the FMT-treated effective group changed from the ineffective group. Among them, *Eubacterium eligens*, *Eubacterium ventriosum*, Clostridiales bacterium, uncultured Blautia sp., Clostridioides difficile, uncultured Clostridium sp., and *Roseburia hominis* correlated with gastrointestinal changes and improvement of PD symptoms.

**Table 3:** Microbial changes after FMT treatment to PD patients.

Author and year	Microbiota detection methods	The domi nant bacteria in the fecal microbiota of PD patients after FMT	The altered microbial species between responders and non responders
Kuai et al. (2021)	16S rDNA sequencing	Coriobacteriaceae, Erysipelotrichaceae and Lachnospiraceae	/
Cheng et al. (2023)	16S rDNA sequencing	/	Eubacterium eligens, *Eubacterium ventriosum*, Clostridiales bac terium 42_27, uncultured *Blautia* sp., Clostridioides difficile, uncultured *Clostridium* sp., and Roseburia hominis
DuPont et al. (2023)	Whole Metagenome Shotgun sequencing	Lactobacillaceae, Limnochordaceae, and Peptostreptococcaceae	/

### 3.5 Preparation and delivery of FMT materials

**Figure 3:**
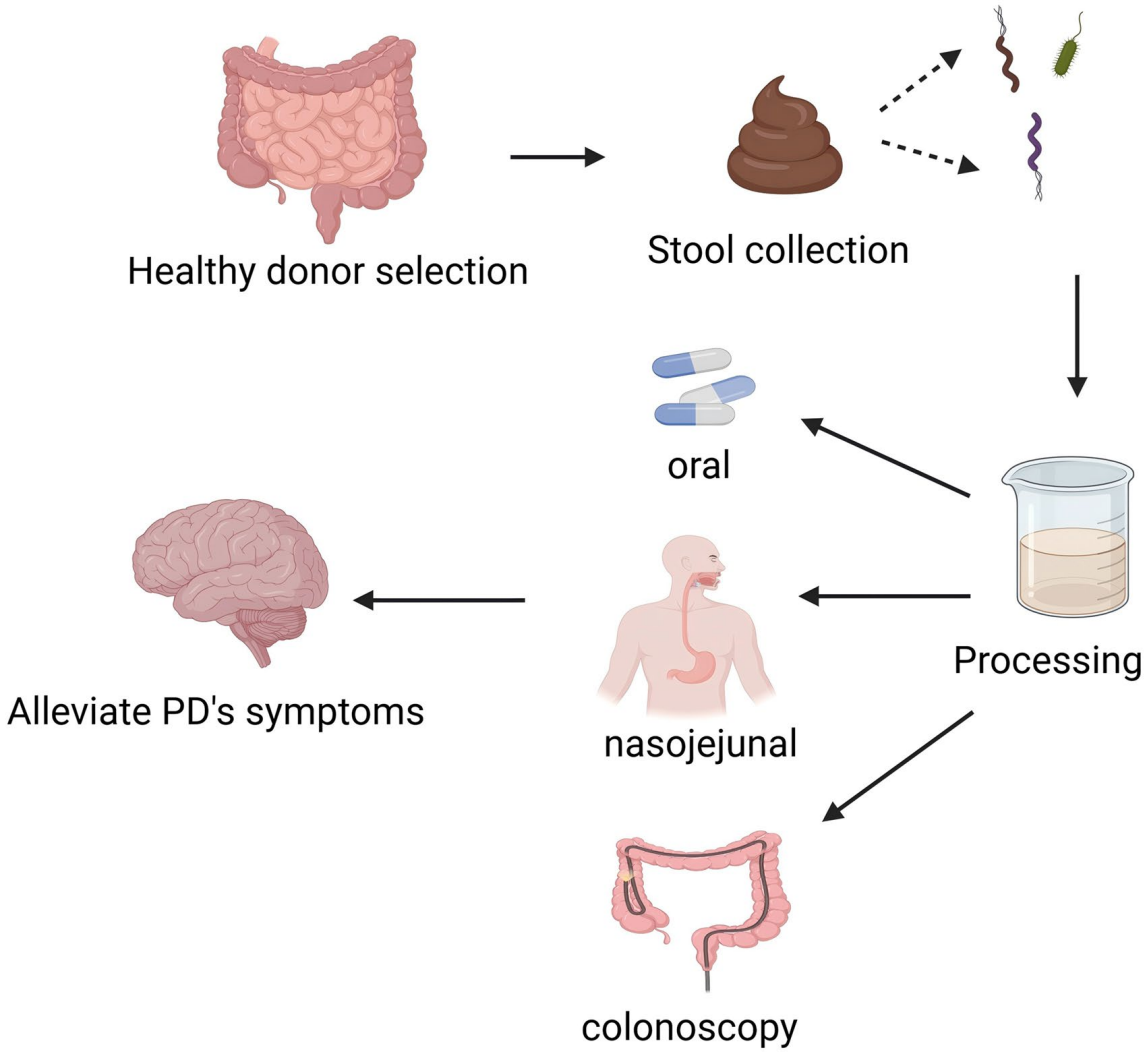
Preparation and procedure and route of administration of FMT. Flowchart illustrating a process starting with healthy donor stool selection. The stool is collected and processed. It can then be administered orally, via nasojejunal tube, or colonoscopy. The aim is to alleviate Parkinson’s disease symptoms, represented by an arrow pointing to a brain.

FMT material from unrelated donors was used in all included studies. Donor fecal samples were collected and processed through multiple stages, including filtration or dilution, to prepare a standardized suspension (Figure 3). Within the selected research, there was a significant diversity in the preparation and administration techniques of the FMT materials (Table 2). Two studies made faeces into capsules (Cheng et al., 2023; DuPont et al., 2023), whereas others used faeces mixed with preservation solution and then frozen and stored (Segal et al., 2021; Kuai et al., 2021; Bruggeman et al., 2024; Scheperjans et al., 2024). FMT materials were delivered to the recipients by oral (Cheng et al., 2023; DuPont et al., 2023) and nasojejunal tube (Kuai et al., 2021; Bruggeman et al., 2024) delivery as well as by colonoscopy [19,24] to recipients (Table 2). Most studies performed only a one-time FMT transplantation, while some studies performed a course of FMT (Cheng et al., 2023; DuPont et al., 2023). Several studies reported that subjects underwent bowel cleansing and colonoscopy to screen for contraindications prior to FMT (Segal et al., 2021; Bruggeman et al., 2024; Scheperjans et al., 2024).

## 4 Discussion

Parkinson’s disease currently lacks novel therapeutic treatments, and FMT has received much attention as a potential treatment (Hamilton et al., 2024). FMT is a method of introducing multiple bacterial families into the diseased gut to restore intestinal diversity (Bakken et al., 2011). This systematic review included four randomized controlled trials, one cohort study and one case study. The overall quality of the studies was high, but could not be quantified due to significant heterogeneity in terms of statistical metrics and follow-up time. Our study suggests that FMT may have a positive effect on improving a wide range of symptoms in patients with PD. This change occurred mainly before and after FMT treatment, whereas this difference was not significant when comparisons with the placebo group were made. This difference may be due to different responsiveness to FMT between individuals. Microbiomics analyses showed that the recovery of the bacterial flora after FMT was characterized by biodiversity, increased stability and significant similarity to donor characteristics. In addition, no serious adverse events occurred. These studies provide a valuable rationale for FMT in patients with PD.

The gut–brain axis is a dynamic, bidirectional communication network linking the gut microbiota to the central nervous system via neuro-immune-endocrine pathways (Naomi et al., 2021). Imbalances in the gut flora influence the onset and progression of neurodegenerative diseases and mental disorders, whereas improving the gut flora can improve various cognitive functions (Barbosa and Vieira-Coelho, 2020; Barrio et al., 2022). Probiotics have been recommended for the management of PD. Several scientific trials have shown that probiotic supplementation improves constipation, bowel habits, stool consistency, and reduces scores on the Parkinson’s Disease Rating Scale (Tamtaji et al., 2019). FMT demonstrates therapeutic efficacy through its regulatory effects on the ‘microbiota-immune-neural’ triad axis, which is crucial for maintaining gut-brain homeostasis (Eslami et al., 2025). Firstly, FMT reshapes the gut microbiota structure by reducing pro-inflammatory bacteria (e.g., Desulfovibrio) and increasing anti-inflammatory bacteria (e.g., Akkermansia, Roseburia), thereby promoting the production of short-chain fatty acids (SCFAs). These SCFAs repair the intestinal barrier by upregulating tight junction proteins (ZO-1, occludin), blocking the translocation of lipopolysaccharides (LPS) into the bloodstream, and consequently inhibiting the microglial TLR4/MyD88/NF-κB inflammatory pathway, which reduces neuroinflammation in the substantia nigra region (Zhao et al., 2021; Sun et al., 2018). Secondly, SCFAs synergize with specific probiotics (e.g., Bifidobacterium) to upregulate tyrosine hydroxylase (TH) expression, increasing dopamine synthesis. Simultaneously, they stimulate enterochromaffin cells via the vagus nerve to release 5-HT and promote the expression of brain-derived neurotrophic factor, thereby restoring neurotransmitter balance and neuronal survival (Bonaz et al., 2018; Church et al., 2023). Ultimately, these mechanisms collectively alleviate the symptoms of the PD model.

Our study suggests that FMT may have a positive effect on improving both motility and non-motility symptoms in PD patients. Specifically, several studies have reported significant reductions in UPDRS and H-Y scores following FMT treatment. In comparative analyses between groups, multiple RCTs demonstrate a better improvement in MDS-UPDRS scores in the FMT group than in the control group. MDS-UPDRS part 3 (off-medication), which assesses motor symptoms, also improved significantly. Constipation has been shown to have a strong association with and intestinal flora, and FMT has been used safely and effectively in the clinical management of functional constipation and inflammatory bowel disease (Ohkusa et al., 2019). This study confirms that most PD patients undergoing FMT will have improved fecal property related scores such as PAC-QOL and Wexner. Several animal and clinical experiments have also confirmed the promoting effect of FMT on neurological recovery (Tao et al., 2025; Wang et al., 2024; Deng et al., 2021; Jing et al., 2021). FMT promotes functional recovery and neuronal axonal regeneration in mice with spinal cord injury (Jing et al., 2021). Some gut bacteria are capable of synthesizing and releasing neurotransmitters such as glutamate, *γ*-aminobutyric acid (GABA) and dopamine, and FMT promotes the restoration of normal levels of neurotransmitters by re-establishing the gut flora (Strandwitz et al., 2019; Wang et al., 2020). The ameliorative effect of FMT on symptoms of depression and fatigue in PD patients has also been confirmed in several studies included in this paper.

In terms of safety, our review found that FMT for PD was well tolerated, with no treatment-related serious adverse events reported. Most of the adverse events were mild self-limiting gastrointestinal reactions, which is consistent with the properties of FMT therapy. FMT is considered a safe and effective treatment modality for a variety of diseases. A meta-analysis of the safety of FMT over a 20-year period showed that the incidence of serious adverse events was only 1.4% (Marcella et al., 2021). These results suggest that FMT has a favorable safety profile in the treatment of PD. However, due to the small sample size, further studies on long-term safety are needed. In the included studies, most of the PD patients received FMT only once, and some took the capsules orally for a consecutive period of time (Cheng et al., 2023; DuPont et al., 2023), but the effects of repeated treatments of FMT versus a single treatment were not compared. While others such as in inflammatory bowel disease found repeated treatments of FMT to be more effective than monotherapy (Goyal et al., 2018; Mocanu et al., 2021; Zou et al., 2023). In a study of FMT for *Clostridium difficile* (CDI), sustained therapeutic efficacy of single FMT treatment reached 50% and CDI cure with repeated FMT reached 84% (Porcari et al., 2023). This may be due to the fact that antigen-specific T cells of the host may destroy the transplanted flora, resulting in ineffective FMT. FMT attenuated the rise in levodopa-equivalent daily dose, consistent with microbiome-encoded tyrosine decarboxylase (tyrDC) converting levodopa to dopamine; higher fecal tyrDC correlates with greater levodopa requirements, highlighting microbial modulation of drug efficacy (Van Kessel et al., 2019; Maini Rekdal et al., 2019). In our review, the donors were mainly unrelated healthy individuals. Emerging evidence indicates that donor–recipient microbiome compatibility strongly influences engraftment success and clinical efficacy; the extent to which donor strains can coexist with, or replace, resident communities is a critical determinant of long-term benefit (Li et al., 2016; Chen et al., 2024). Methods of preparation and delivery of FMT materials have also varied across studies, which may affect therapeutic efficacy. In this paper’s studies, FMT material primarily took the form of capsules and cryopreserved feces, with oral, nasoenteric, and colonoscopy being the primary delivery methods. The studies did not analyze the impact of FMT materials on PD outcomes. One study compared oral capsule and colonoscopic FMT for RCDI treatment, finding oral capsules to be as effective as colonoscopy in achieving clinical remission (Kao et al., 2017). Oral capsule FMT provides a simple and convenient route of administration for clinicians and patients (Allegretti et al., 2020). However, emerging encapsulated FMT products vary in formulation due to a lack of standardized methods, and these differences may have an impact on therapeutic efficacy (Halaweish et al., 2022). Therefore, standardized methods for the preparation and delivery of FMT materials are essential to improve therapeutic efficacy.

Post-FMT microbiome alterations are linked to PD symptom improvements, as gut bacteria generate metabolites like SCFAs from fermentation and modify host molecules such as bile acids (BAs), in addition to direct bacterial products (Paramsothy et al., 2019; Collins et al., 2023). SCFAs can stimulate neurotransmitter synthesis in central and peripheral systems (Zhong et al., 2023) and resist neuronal apoptosis (Xiao et al., 2022). Altered secondary BAs by gut microbiota inversely relate to depression severity. FMT’s rectification of gut dysbiosis may yield metabolites that slow brain disease progression. Our study observed that FMT altered gut flora diversity and composition in PD patients, correlating with better clinical outcomes, underscoring the role of MGBA in PD and justifying FMT’s microbiological basis. We also noted reduced species diversity and distinct microbiome profiles in PD patients versus controls. And after FMT such as Roseburia, Colinsella, and Faecalibacterium became the predominant genera after FMT treatment. Analyses for both FMT and control groups revealed significant abundance of, e.g., Lactobacillaceae, Limnochordaceae and Peptostreptococcaceae in the FMT treated group. Notably, clinical responders to FMT had higher species diversity and higher abundance of some specific flora compared to non-responders than the FMT non-responder group. A meta-analysis that included multiple national cohorts reported that intestinal mucin layer-degrading Akkermansia is increased in patients with PD and that short-chain fatty acid-producing Roseburia and Faecalibacterium are decreased (Nishiwaki et al., 2020). The FMT clinical cohort included in the present study increased exactly these beneficial flora that are deficient in the gut of PD patients. Cignarella et al. (2018) reported that intermittent fasting induced an increase in Lactobacillaceae that had a protective effect against Multiple sclerosis. An increase in Lactobacillaceae was also found in the FMT group compared to the placebo group in the present study, which demonstrates the effectiveness of FMT treatment. Vaughn et al. (2023) research indicated that FMT responders experienced an upregulation of several metabolic pathways, such as serine and glutamine metabolism, folate metabolism, and lipid A biosynthesis, compared to non-responders. Aligning with our findings, the enrichment of diverse flora in FMT clinical responders relative to non-responders could be pivotal for clinical symptom improvement.

To move the field forward, large-scale, multicenter randomized trials are urgently needed. We propose the following key elements for such studies: (1) Donor screening should follow a harmonized protocol (age 18–45 y, BMI 18–25, validated questionnaires plus negative stool PCR for enteric pathogens and parasites, and absence of antibiotic exposure within 3 months). (2) FMT material should be standardized to ≥50 g fresh stool equivalent per dose, prepared as either frozen–thawed suspension or enteric-coated capsules with a uniform cryopreservation/thawing SOP. (3) Primary endpoint: change in MDS-UPDRS total score at 24 weeks; secondary endpoints should include H-Y stage, NMSS, PAC-QOL/Wexner score, LEDD, and gut microbiome profiling (16S or shotgun metagenomics) at baseline, 4, 12 and 24 weeks. (4) Study design: randomized, double-blind, placebo-controlled, minimum 200 participants per arm, stratified by disease duration (<5 vs. ≥ 5 years) and baseline constipation severity. Establishing a centralized biobank and data-coordinating center will ensure protocol fidelity across sites.

In summary, FMT, as an emerging therapeutic tool, has shown some efficacy and a favorable safety profile in the treatment of PD. Nevertheless, the small sample sizes and absence of stratified reporting in the included studies hinder our understanding of whether FMT efficacy differs across clinically relevant subgroups. Future studies need to adopt larger sample sizes, pre-stratified randomization (by PD subtype, disease duration and baseline constipation severity), and harmonized outcome measures to further validate the role of FMT in PD treatment. A key limitation is that the review protocol was neither pre-registered nor designed under the Synthesis Without SWiM guideline. Consequently, decisions on study grouping, standardized metrics and heterogeneity exploration were made *post hoc*, potentially introducing subjective bias and reducing reproducibility. Because fewer than 10 studies were eligible, funnel-plot assessment of publication bias was not feasible. Moreover, given the multifactorial pathogenesis of Parkinson’s disease and the complexity of the MGBA, future studies should systematically evaluate multimodal regimens that combine FMT with (1) targeted probiotics or prebiotics administered sequentially to facilitate donor-microbiota engraftment and prolong efficacy, (2) low-dose anti-inflammatory agents such as 5-ASA or IL-10 inducers to synergistically suppress neuroinflammation, and (3) conventional medications such as levodopa to reduce dosing requirements and side-effects, thereby maximizing the therapeutic potential of FMT and advancing precision micro-ecological interventions.

## Data Availability

The original contributions presented in the study are included in the article/Supplementary material, further inquiries can be directed to the corresponding author/s.

## Supplementary material

Supplementary Table 1 Quality assessment of controlled, interventional studies.

Supplementary Table 2 Quality assessment of cohort studies.

Supplementary Table 3 Quality assessment of case-series.
