# Supplementary material for: Safety and efficacy of fecal microbiota transplantation in the treatment of Parkinson’s disease: a systematic review of clinical trials
Source: Front Neurosci. 2025 Aug 20;19:1639911. doi: 10.3389/fnins.2025.1639911 (PMC12405258; doi:10.3389/fnins.2025.1639911)
Supplement: Supplementary file 5 [file Data_Sheet_1.pdf]

Pubmed

1 "Parkinson Disease"[Mesh] Results:89318

2 ("Parkinson Disease"[Mesh]) OR (parkinson*[Title/Abstract]) Results:169758

3 "Fecal Microbiota Transplantation"[Mesh] Results:3658

4 ((((((((((((("Fecal Microbiota Transplantation"[Mesh]) OR (Fecal Microbiota Transplantations[Title/Abstract])) OR (Transplantation, Fecal Microbiota[Title/Abstract])) OR (Transplantations, Fecal Microbiota[Title/Abstract])) OR (Fecal Microbiota Transplant[Title/Abstract])) OR (Fecal Microbiota Transplants[Title/Abstract])) OR (Transplant, Fecal Microbiota[Title/Abstract])) OR (Transplants, Fecal Microbiota[Title/Abstract])) OR (Fecal Microbiome Transplantation[Title/Abstract])) OR (Fecal Microbiome Transplantations[Title/Abstract])) OR (Fecal Transplant[Title/Abstract])) OR (Fecal Transplants[Title/Abstract])) OR (Donor Feces Infusion[Title/Abstract])) OR (Donor Feces Infusions[Title/Abstract]) Results:6952

5 ((((((((((((((("Fecal Microbiota Transplantation"[Mesh]) OR (Fecal Microbiota Transplantations[Title/Abstract])) OR (Transplantation, Fecal Microbiota[Title/Abstract])) OR (Transplantations, Fecal Microbiota[Title/Abstract])) OR (Fecal Microbiota Transplant[Title/Abstract])) OR (Fecal Microbiota Transplants[Title/Abstract])) OR (Transplant, Fecal Microbiota[Title/Abstract])) OR (Transplants, Fecal Microbiota[Title/Abstract])) OR (Fecal Microbiome Transplantation[Title/Abstract])) OR (Fecal Microbiome Transplantations[Title/Abstract])) OR (Fecal Transplant[Title/Abstract])) OR (Fecal Transplants[Title/Abstract])) OR (Donor Feces Infusion[Title/Abstract])) OR (Donor Feces Infusions[Title/Abstract])) AND (("Parkinson Disease"[Mesh]) OR (parkinson*[Title/Abstract])) Results:133

Embase

1 'parkinson disease'/exp Results:214885

2 parkinson*:ti,ab,kw Results:233837

3 #2 OR #3 Results:274315

4 'fecal microbiota transplantation'/exp Results:12738

5 'transplantation, fecal microbiota':ti,ab,kw OR 'transplantations, fecal microbiota':ti,ab,kw OR 'fecal microbiota transplant':ti,ab,kw OR 'transplant, fecal microbiota':ti,ab,kw OR 'transplants, fecal microbiota':ti,ab,kw OR 'fecal microbiome transplantation':ti,ab,kw OR 'fecal transplant':ti,ab,kw OR 'donor feces infusion':ti,ab,kw Results:1621

6 #4 OR #5 Results:13038

7 #3 AND #6 Results:345

Web of science

1 TS=(Parkinson Disease) OR TS=(parkinson*) Results:389948

2 TS=(Fecal Microbiota Transplantation) OR TS=(Transplantation, Fecal Microbiota) OR TS=(Fecal Microbiota Transplant) OR TS=(Transplant, Fecal Microbiota) OR TS=(Fecal Microbiome Transplantation) OR TS=(Fecal Transplant) OR TS=(Donor Feces Infusion) Results:13699

3 #2 AND #1 292

Scoups

1 (TITLE-ABS-KEY ("Parkinson Disease") OR TITLE-ABS-KEY ("parkinson*")) Results:246,892

2 (TITLE-ABS-KEY ("Fecal Microbiota Transplant*") OR TITLE-ABS-KEY ("Transplant*, Fecal Microbiota") OR TITLE-ABS-KEY ("Fecal Microbiome Transplant*") OR TITLE-ABS-KEY ("Fecal Transplant*") OR TITLE-ABS-KEY ("Donor Feces Infusion")) Results:12,519

3 ((TITLE-ABS-KEY ("Fecal Microbiota Transplant*") OR TITLE-ABS-KEY ("Transplant*, Fecal Microbiota") OR TITLE-ABS-KEY ("Fecal Microbiome Transplant*") OR TITLE-ABS-KEY ("Fecal Transplant*") OR TITLE-ABS-KEY ("Donor Feces Infusion"))) AND ((TITLE-ABS-KEY ("Parkinson Disease") OR TITLE-ABS-KEY ("parkinson*"))) Results:377
